# Processing of Visual Speech Cues in Speech-in-Noise Comprehension Depends on Working Memory Capacity and Enhances Neural Speech Tracking in Older Adults With Hearing Impairment

**DOI:** 10.1177/23312165241287622

**Published:** 2024-10-24

**Authors:** Vanessa Frei, Raffael Schmitt, Martin Meyer, Nathalie Giroud

**Affiliations:** 1 Computational Neuroscience of Speech and Hearing, Department of Computational Linguistics, 27217 University of Zurich, Zurich, Switzerland; 2 International Max Planck Research School for the Life Course: Evolutionary and Ontogenetic Dynamics (LIFE), Berlin, Germany; 3 Competence Center Language & Medicine, Center of Medical Faculty and Faculty of Arts and Sciences, University of Zurich, Zurich, Switzerland; 4 27217 University of Zurich, University Research Priority Program Dynamics of Healthy Aging, Zurich, Switzerland; 5 587553 Center for Neuroscience Zurich, University and ETH of Zurich, Zurich, Switzerland; 6 Evolutionary Neuroscience of Language, Department of Comparative Language Science, 27217 University of Zurich, Zurich, Switzerland; 7 Cognitive Psychology Unit, 172150 Alpen-Adria University, Klagenfurt, Austria

**Keywords:** neural speech tracking, audio-visual speech, age-related hearing loss, EEG, working memory capacity, speech in noise

## Abstract

Comprehending speech in noise (SiN) poses a challenge for older hearing-impaired listeners, requiring auditory and working memory resources. Visual speech cues provide additional sensory information supporting speech understanding, while the extent of such visual benefit is characterized by large variability, which might be accounted for by individual differences in working memory capacity (WMC). In the current study, we investigated behavioral and neurofunctional (i.e., neural speech tracking) correlates of auditory and audio-visual speech comprehension in babble noise and the associations with WMC. Healthy older adults with hearing impairment quantified by pure-tone hearing loss (threshold average: 31.85–57 dB, *N* = 67) listened to sentences in babble noise in audio-only, visual-only and audio-visual speech modality and performed a pattern matching and a comprehension task, while electroencephalography (EEG) was recorded. Behaviorally, no significant difference in task performance was observed across modalities. However, we did find a significant association between individual working memory capacity and task performance, suggesting a more complex interplay between audio-visual speech cues, working memory capacity and real-world listening tasks. Furthermore, we found that the visual speech presentation was accompanied by increased cortical tracking of the speech envelope, particularly in a right-hemispheric auditory topographical cluster. Post-hoc, we investigated the potential relationships between the behavioral performance and neural speech tracking but were not able to establish a significant association. Overall, our results show an increase in neurofunctional correlates of speech associated with congruent visual speech cues, specifically in a right auditory cluster, suggesting multisensory integration.

## Introduction

Difficulty understanding spoken language, particularly in challenging listening situations, is common with aging (Humes et al., 2012; Zekveld et al., 2011). Concurrently, age-related changes in the cochlear structure, such as a decrease or damage in the number of outer hair cells and therefore reduced transduction of acoustic energy within the inner ear (Ashmore, 2008; Dubno et al., 2013; Mills et al., 2006) partially contribute to elevated pure-tone thresholds in the high-frequency range. These cochlear deficits often result in pure-tone hearing loss, which is highly prevalent in older age (Homans et al., 2017). Nevertheless, pure-tone hearing loss does not seem to fully account for SiN perceptual difficulties. This is indicated by individuals who have matching audiograms and who still vary significantly in their SiN perceptual performance (Anderson et al., 2011; Vermiglio et al., 2012). Given such inter-individual variability in SiN perceptual abilities in older adults with similar audiograms, varying cognitive capacity should also be considered as a contributing factor (Humes et al., 2012). Cognition, particularly working memory, appears to partly bridge the gaps in auditory input created by reduced audibility, supporting sensory processing and compensating for reduced spectro-temporal precision (Anderson et al., 2013; Pichora-Fuller & Souza, 2003; Wong et al., 2009, 2010). This rationale aligns with a theory of an integrated perceptual-cognitive system (Schneider & Pichora-Fuller, 2000), which assumes that a shared pool of resources is available for cognitive as well as perceptual demands.

Also, on the neural level, there is individual variability in how speech in noise is being processed in auditory-related areas. In general, while listening to natural continuous speech in quiet or in noise, a synchronization between low-frequency activity in the auditory cortex and temporal regularities of the speech signal can be observed (e.g., Ding & Simon, 2013). Such phase-locking of the neural response, particularly to the amplitude envelope, is often referred to as neural tracking of speech (Luo & Poeppel, 2007). It is assumed that the quasi-rhythmic nature of natural speech might be associated with neural speech tracking, reflecting cortical encoding of speech by segmenting the continuous stream into discrete acoustic units, facilitating higher-order acoustic and linguistic encoding of speech features (Ghitza, 2013; Giraud & Poeppel, 2012; Poeppel & Assaneo, 2020, for other proposals see Ding & Simon, 2014). There is still a debate surrounding the question, of whether neural speech tracking has a functional role for speech understanding. Some studies report a positive relationship between a stronger neural response (e.g., higher degree of synchronization between neural activity and speech envelope) and speech in noise perception in older individuals with and without hearing impairment (e.g., Decruy et al., 2019; Schmitt et al., 2022), while others report the opposite relationship, namely a decrease in speech understanding with enhancing neural speech tracking (e.g., Goossens et al., 2018; Millman et al., 2017). On the one hand, it is argued that an increased tracking response might reflect more precise processing of the target speech due to degradation of the signal (e.g., through hearing loss or noise). On the other hand, this enhanced tracking response might reflect an overrepresentation of the speech signal in the brain due to insufficient inhibition (e.g., Presacco et al., 2016) or an inefficient processing mechanism due to reduced cortical connectivity (Peelle & Sommers, 2015). This debate highlights the importance of considering neural speech processing in the context of SiN perceptual difficulties.

In addition to the question of the functional role of neuronal speech tracking, there is also a need to discuss the localization and lateralization of speech processing in the brain of older people with hearing impairment. Poeppel (2003) suggested that the initial representation of speech is bilateral in auditory-related areas (Hickok & Poeppel, 2007) but is processed asymmetrically over time. Information from short temporal chunks (20–40 ms) is processed more pronounced in the left auditory regions and longer temporal windows (150–250 ms) in the right auditory regions. However, this asymmetric sampling in time (AST) hypothesis relates to younger individuals with age-typical auditory processing. Giroud et al. (2019) extend the AST model through the perspective of aging. Following Poeppel et al. (2007), they argue that older individuals involve bilateral auditory regions to counteract age-related neurostructural decline (Giroud et al., 2019) and maintain sensitivity to different speech cues and temporal windows.

While many points are still to be addressed regarding the functional role of neural speech processing and speech understanding in age-related hearing loss, there is also an interest in the potential supporting role of audio-visual speech presentation. In a natural conversation, listeners often find themselves in an audio-visual setting, where the speaker's face and mouth movements are visible. Accordingly, the interest in investigating the relationship between speech in noise perception, neural speech processing and audio-visual speech presentation is high. While it is generally established that visual speech cues can improve speech perception, there is a common report of large individual differences in the extent of this benefit. Several studies suggest that older adults with high-frequency hearing loss in particular benefit from visual speech cues and show improved SiN performance (Altieri & Hudock, 2014; Hallam & Corney, 2014; Lidestam et al., 2014; Winneke & Phillips, 2011), while other studies find a benefit to speech perception from audio-visual speech presentation, but independent of age and the degree of hearing loss and with considerable individual variability in the extent of this benefit (Başkent & Bazo, 2011; Rosemann & Thiel, 2018; Sommers et al., 2005; Tye-Murray et al., 2007). At the neural level, visual speech cues appear to enhance neural tracking of the speech envelope (Aller et al., 2022; Crosse et al., 2016; Micheli et al., 2018; Park et al., 2016) and restore early cortical tracking of speech presented in noise, complementing impaired auditory input (Atilgan et al., 2018; Crosse et al., 2015; Zion Golumbic et al., 2013). While enhanced neural speech tracking can be observed under congruent visual speech cues, it is necessary to establish if this increase is simply the addition of two unimodal streams of information or rather a bimodal integration (for an overview of assessment methods for multimodal integration see Stevenson et al., 2014). A frequently used approach is to test the presence of a difference between the audio-visual response and the algebraic sum of the audio-only and the visual-only response, whereby a difference is argued to reflect multisensory integration (e.g., Peelle, 2019). This audio-visual increase of neural speech tracking has also been observed in older individuals (Puschmann et al., 2019). The authors report a significant increase in envelope tracking in the presence of congruent visual speech cues. Furthermore, envelope tracking increased with increasing levels of subjectively reported listening effort. While this work demonstrates multisensory integration in the context of older hearing-impaired individuals, the authors do not establish a relationship between the extent of neural speech tracking and behavioral speech perception.

Thus, this study aims to investigate the extent to which visual speech cues facilitate speech perception in babble noise in a large sample of older adults (*N* = 67) with varying degrees of pure-tone hearing loss and cognitive capacity. More specifically, the relationship between neural speech tracking and SiN pattern matching (whereby participants have to decide if a sound snipped was part of the before-heard sentence or not), and comprehension performance will be investigated, in auditory as well as audio-visual speech presentation. The current study reports on speech in babble noise perception in varying speech presentation modalities, reflecting a part of a bigger data collection and adding important evaluations to the work conducted by Schmitt et al. (2022), who investigated speech processing within partially the same sample, focusing on varying noise conditions. We hypothesize that audio-visual speech presentation is associated with improved SiN pattern matching and comprehension in older individuals with hearing impairment compared to auditory-only speech exposure. In addition, we hypothesize that working memory capacity explains some of the individual variability in SiN pattern matching and comprehension. Furthermore, we hypothesize that the neural response is altered in the audio-visual speech presentation modality such that neural speech tracking is greater in the audio-visual modality than in the auditory-only modality. Lastly, we assume that increased neural speech tracking is associated with better SiN pattern matching and comprehension.

## Materials and Methods

### Sample

The study included 67 healthy older participants (*M*_Age _= 72, Range_Age _= 64–80, *SD*_Age _= 4.3, male = 41) and did not show any cognitive impairments (Montreal Cognitive Assessment > 26 points; Nasreddine et al., 2005), pre-existing neurological or psychiatric conditions, speech or language disorders. They were not professional musicians and had sufficient or corrected-to-normal vision. The participants were all native speakers of Swiss German and had not learned a second language before the age of seven. Additionally, pure-tone hearing loss did not exceed 60 dB HL for octave frequencies between 0.5 and 8 kHz (*M*_PTA _= 42.58, Range_PTA _= 31.85–57, *SD*_PTA _= 6.33) and pure-tone averages (PTA) were nearly symmetrical for both ears (<15 dB interaural threshold difference). The sample was split into two groups, with 34 participants having experience using hearing aids (HA) for at least 12 months and 33 had never used hearing aids (nHA). Both groups were included in the study to represent a broad range of older individuals with hearing loss, no significant differences were found between the two groups in cognitive abilities, age, or hearing loss (Table 1). The hearing-aid users completed the study (including audiometry) with their hearing aids in to represent their daily auditory experience. As mentioned before, the here presented data were generated in a larger study design, containing additional speech presentation conditions and participants. The current study only focused on participants with mild to moderate hearing, while the study by Schmitt et al. (2022) included individuals without hearing loss, excluding participants with hearing aids. All participants provided written informed consent and were compensated for their participation. The study was conducted ethically, in compliance with the Declaration of Helsinki and approved by the local ethics committee (Cantonal Ethics Committee Zurich, application no. 2017-00284).

**Table 1.** Comparability Between Individuals With and Without Hearing Aids Regarding Age, Hearing Loss, and Working Memory Capacity.

	HA (*n* = 34)		nHA (*n* = 33)			
Assistance	*M*	*SE*	*M*	*SE*	*t*(65)	*p*
Age	72.97	4.15	71.12	4.42	1.765	.082
PTA	43.22	6.75	42.13	6.28	0.686	.495
WMC	49.35	8.79	49.33	8.76	0.009	.993

### Audiometry

**Figure 1.**
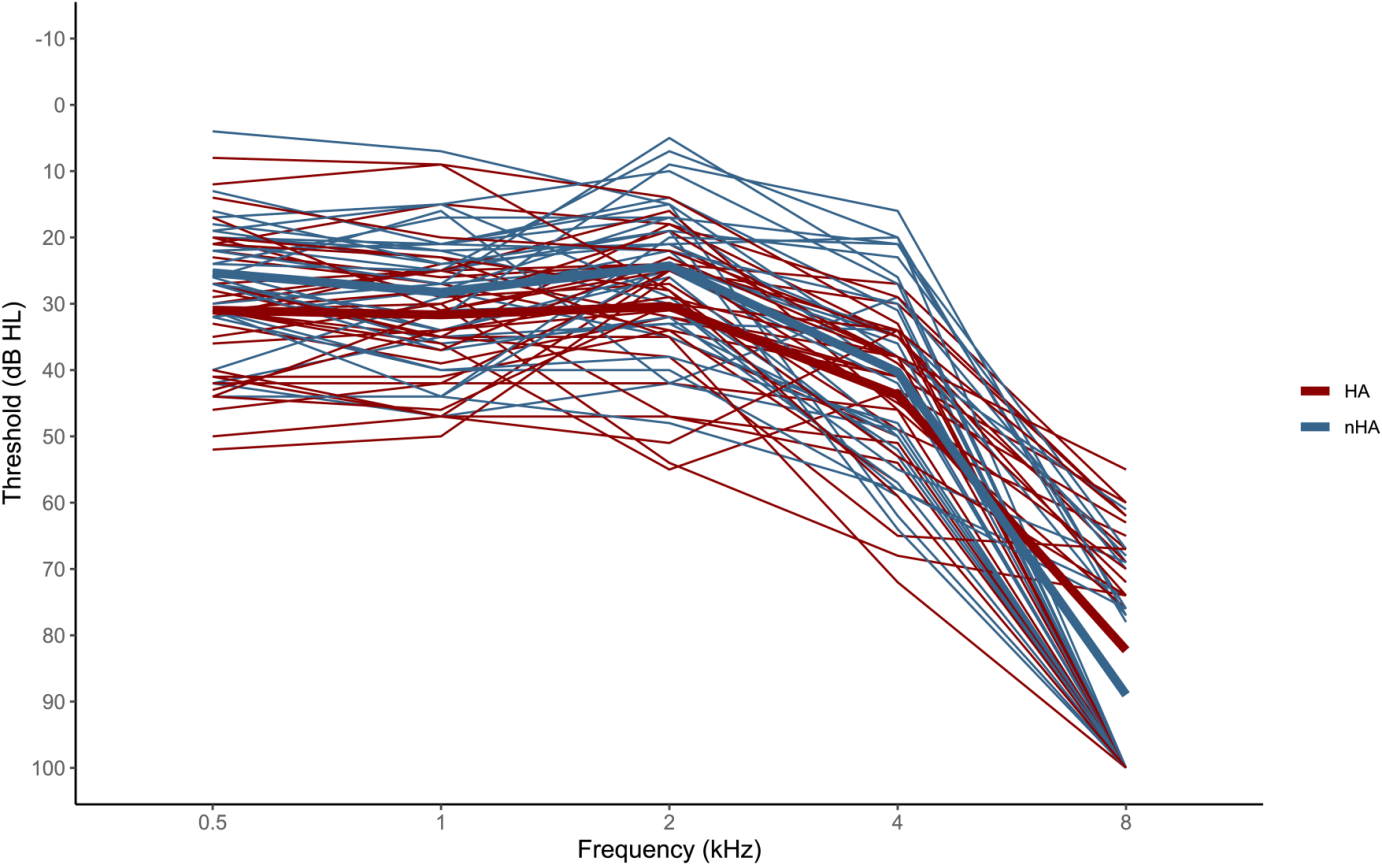
**Pure-tone audiometry**. The audiogram depicts individual pure-tone thresholds at frequencies between 0.5 and 8 kHz. There is no systematic difference between hearing-aid users (HA; group average depicted in red) and non-hearing-aid users (nHA; group average depicted in blue). Stimulus presentation was limited to 100 dB, which explains the accumulation of data points at 8 kHz. Hearing-aid users were measured while having their devices on.

To determine participants’ hearing loss, pure-tone thresholds were measured using a MATLAB-based probe-detection paradigm that has been described in detail in previous studies (Giroud et al., 2018; Lecluyse & Meddis, 2009). Stimuli were controlled via a sound card (RME Babyface Pro, RME, Haimhausen, Germany) and delivered through a linear frequency response loudspeaker (8030B Studio Monitor, Genelec, Iisalmi, Finland), with participants seated in an electrically shielded soundproof booth. Hearing-aid users were measured with their devices in. After the measurement, the PTA was calculated by averaging the individual thresholds over the frequencies of 0.5, 1, 2, 4, and 8 kHz. The HA users were instructed to wear their hearing aid throughout the audiometry as well, to ensure that their everyday auditory experience was comparable to the nHA group. As can be seen in the audiogram (Figure 1), across both groups pure tone hearing becomes difficult in the high frequency regions (4 kHz and 8 kHz). Hearing acuity shows a large variability across individuals, but not across groups (HA vs, nHA). Audiograms are visualized in Figure 1.

### Stimuli and Experimental Set-up

The current study reflects two speech presentation modalities, namely auditory and audio-visual speech presentation, while the presented speech was accompanied by multi-talker babble noise, which is described in detail in the following sections. However, these conditions are only part of a bigger data collection, consisting of varying noise conditions and modalities. The overall experimental procedure consisted of an initial screening session (t0), whereby hearing acuity (detailed description under audiometry) a tinnitus screening as well as screening for mild cognitive impairment (Montreal Cognitive Assessment, German Version, Nasreddine et al., 2005) and general health and demographic questionnaires were assessed. A second appointment (t1) was used for a structural MRI measurement, whereby anatomical t1 weighted MR images were conducted. And the third appointment (t2) was used to assess a cognitive test battery followed by the EEG paradigm. The cognitive test battery took roughly 90 min and consisted of word fluency (Mehrfachwahl-Wortschatz-Intelligenztest MWB-T: Lehrl, 1992), processing speed (Kurztest Allgemeine Intelligenz KAI: Lehrl et al., 2016), executive functions & attention (Flanker Task and Go-/No-Go task from the Test of Attentional Performance, TAP: Leclercq & Zimmermann, 2004), perceptual speed (Digit symbol coding from Hamburg-Wechsler Intelligenztest für Erachsene: HAWIE-R: Tewes, 1991), working memory (n-back and reading span from TAP) as well as word-fluency (from Leistungsprüfsystem LPS: Horn, 1983). During the EEG paradigm, natural continuous speech was presented in multi-talker babble noise, once in an auditory- and once in an audio-visual speech presentation modality, masked by pink noise (1/f), presented in quiet and for a control condition, a lip-only presentation modality was chosen. Within each condition, a total of 30 sentences were presented, while participants were randomly assigned to 6 different orders of conditional blocks to control for potential fatigue effects. The total duration of t2 was approximately 3 h (including breaks, and 128-channel EEG montage). Several studies have been published describing these additional conditions in detail.

**Figure 2.**
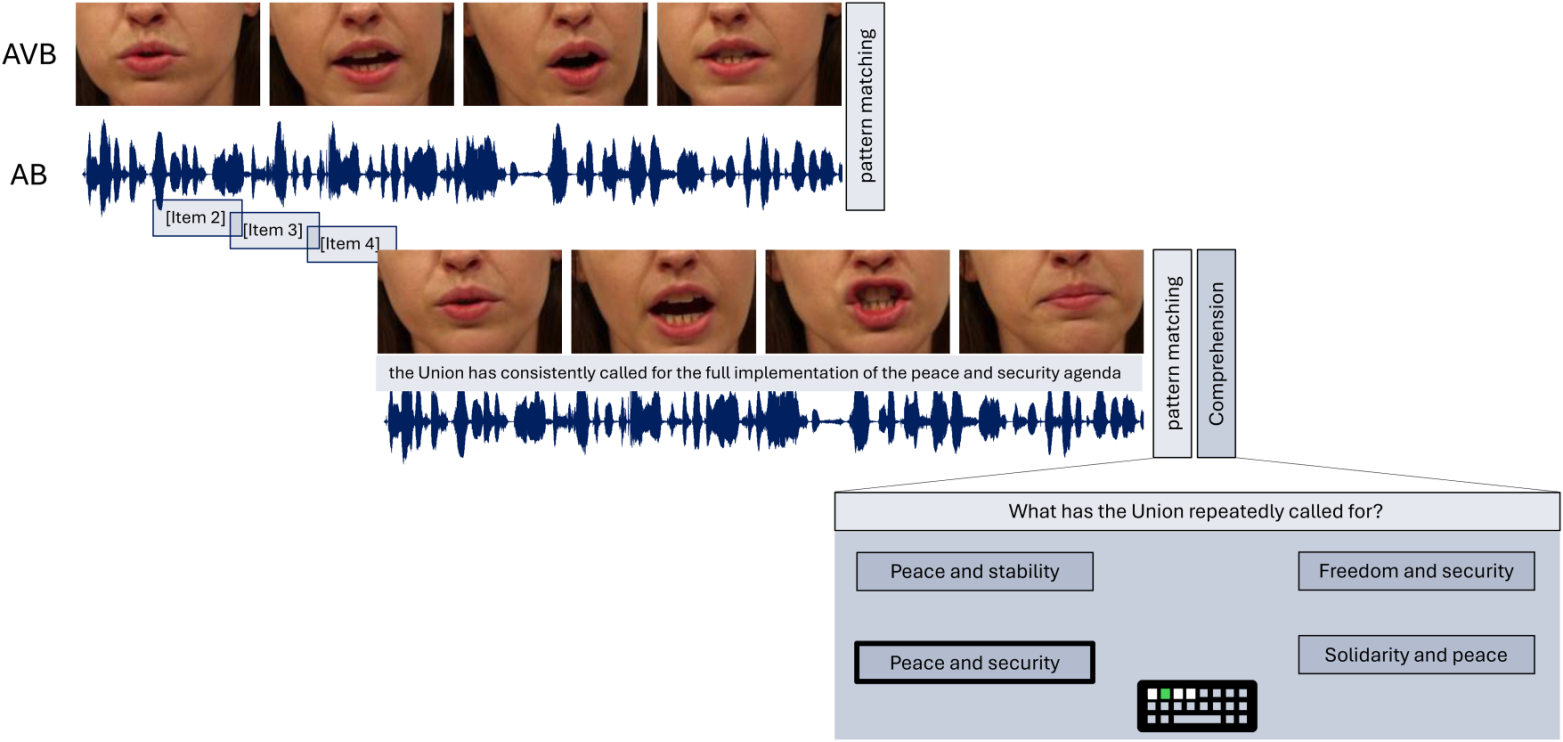
**Illustration of the stimulus presentation**. The modalities differed in that audio-visual-babble (AVB) included a video sequence of the mouth and jaw movement whereas the audio-babble (AB) modality only contained auditory stimuli. Five sentences were presented, and after each, a pattern-matching task was applied. After every fifth sentence, a comprehension question was asked. There was a total of 30 items per modality.

Schmitt et al. (2022) have described the effects of varying noise conditions and hearing-loss. While the current study analyses a considerable overlap of participants, the authors considered a larger range of hearing acuity for their investigation, particularly individuals without hearing loss, while excluding individuals with hearing aids for their sample. Furthermore, the study by Schmitt and colleagues only investigated speech processing in the context of varying noise and quiet, while the current study focused on speech in babble noise presented in varying modalities. While there are considerable deviations between the two studies, it is important to note that the work reported in the current study might pose an additional analysis. Additionally, two other studies have been published, reporting on neuroanatomical correlates in the context of hearing loss and tinnitus (Elmer et al., 2023), as well as neuroanatomical features for temporal and spectral aspects in speech in noise recognition (Neuschwander et al., 2023). The current study focused on the effect of presenting natural continuous speech with and without congruent visual speech cues while also accounting for multi-talker babble noise. Participants were exposed to sentences in standard German by a spoken-trained actress, with an average speech rate of 4.66 syllables per second (ranging from 4.03 to 5.61). The speech rate was determined using the De Jong and Wempe (2009) algorithm for syllable nuclei detection in Praat (version 6.1.40; Boersma & Weenink, 2021). The sentences were designed to have neutral content related to transportation regulations in the European Union, minimizing the activation of existing knowledge. The sentences were presented in two modalities, once in a pure auditory modality (AB) and once in an audio-visual modality (AVB), both accompanied by multi-talker babble noise. In AVB, the speaker's lip movements and the lower half of the face were visible and synchronized with the speech. Additionally, the participants were exposed to a visual-only (VO) modality, whereby the visual speech cues from AVB were presented, but without auditory information, serving as a control condition for multisensory integration. Participants were instructed to watch the mouth movement of the speaker as closely as possible. For the VO condition, the same number of sentences (30 in total) were presented as for the auditory and the audio-visual condition. The babble noise included eight additional randomly selected sentences spoken by the same speaker, while silent pauses were removed to prevent participants from “hearing in the gaps”. Each modality consisted of 30 sentences, with an average duration of 10.34 s (ranging from 8.41 to 12.35 s), at a sound pressure level (SPL) of 70 dB, ensuring that the sentences were audible to all participants. Stimuli were controlled via a sound card (RME Babyface Pro, RME, Haimhausen, Germany) and presented through a loudspeaker with linear frequency response (8030B Studio Monitor, Genelec, Iisalmi, Finland). The signal-to-noise ratio (SNR) for the babble noise was set to 0, based on a pilot study that showed optimal performance at that SNR level. The noise started playing 1.5 s after the onset of the target sentence, allowing participants to focus on the target signal. For AVB, participants were asked to place their chin on a headrest, ensuring an approximate distance between the participant and the screen of 60 cm. Video stimuli were presented in the center of the screen with a display width of 600 pixels and a display height of 400 pixels. The visual experimental stimuli were presented on an HDMI Asus VS228H LCD Monitor. The video showed the speaker's nose, mouth and chin area which is displayed in Figure 2.

Following each sentence, a pattern-matching task was conducted. Participants were presented with a 300-ms sound snippet and were required to indicate whether the snippet belonged to the sentence they had just heard. They provided their response by clicking either the left mouse button (for “yes”) or the right mouse button (for “no”). To minimize the cognitive load, the snippets were randomly extracted from the last 3 to 0.3 s of each sentence. Additionally, after every fifth sentence, a comprehension question with four answer options was displayed on the screen as a four-alternative forced-choice task. Participants selected their responses by clicking the corresponding number keys “1”, “2”, “3”, or “4” on the keyboard to indicate the correct answer. To familiarize participants with the tasks and ensure a clear understanding, the experiment began with a training session conducted in a quiet environment. After each trial, feedback was provided on the screen, and the training trial was repeated until the participant performed without any mistakes. This process ensured that the participants correctly grasped the task requirements. No behavioral tasks were assessed during the VO modality.

### Working Memory Capacity

A computerized n-back task was used, which is part of the Test of Attentional Performance assessment battery, short TAP (Leclercq & Zimmermann, 2004). The n-back paradigm visually presented digits that appeared in rapid succession on the screen. Participants were asked to press a key if the displayed digit had also been displayed two items/digits prior to the current one. Notably, participants did not have to press a key if the presented item did not match the one which was two items prior. While the n-back task is frequently used to probe working-memory functions (Owen et al., 2005), it is important to highlight that this task does not fully reflect working memory capacity, but rather updating and maintaining information. Nevertheless, these specific aspects of working memory are important regarding speech perception, particularly in the context of natural speech, which is why a sequential n-back task was used to investigate if certain aspects of working memory capacity had an influence on speech in noise perception. For the sake of simplicity, the term working memory capacity is used in the following, even if only some aspects of it are quantified using the n-back task. As described above, a cognitive test battery was assessed before the EEG paradigm, providing varying measures for cognitive domains. However, we decided to focus on the performance measured by the n-back task, as this was the best match for the presented tasks.

The completion of the assessment took a total of about 5 min. Instructions were given in standard German. T-scored n-back accuracy was used as a primary indicator for working memory capacity (WMC).

### EEG Recording and Pre-processing

A continuous EEG was recorded with a 128 Ag/AgCl scalp electrode cap (BioSemi ActiveTwo, Amsterdam, The Netherlands) at a sampling rate of 512 Hz, while an online bandpass filter between 0.1 and 100 Hz was applied. The impedances across all electrodes were kept below 25 kΩ. For the preprocessing pipeline, we used the Fieldtrip toolbox (Oostenveld et al., 2011), in MATLAB (R2021a, Mathworks). According to the respective trials, the continuous EEG signals were segmented into smaller units, covering the presented sentence with a 2 s baseline before sentence onset. The preprocessing pipeline started by re-referencing the data to Cz, while a bandstop-filter was applied between 49–51 Hz to control for potential artifacts resulting from electrical interference. In a next step, the segmented data were bandpass filtered between 0.1 and 30 Hz, while we visually inspected and excluded potential bad channels. Independent component analysis (ICA) was applied (Jung et al., 2000), while beforehand data was re-referenced to the average reference. Through ICA we visually inspected components based on time course and topography and identified those reflecting eye blinks and cardiac activity and subsequently removed them from the data. Interpolation of noisy channels was done by applying spherical spline interpolation. Data was then resampled to 128 Hz, bandpass filtered between 2 and 8 Hz and baseline corrected. In a last step, the pre-processed segments were cut into 5-s-long uniform trials (starting at 3 s after speech onset up to 8 s), to control for sentence and noise onset (1.5 s after sentence onset).

### Envelope Extraction

The amplitude envelope corresponding to the presented speech material was preprocessed by the use of a gammatone filterbank (Biesmans et al., 2017) before extracting them through full-wave rectification and power-law compression. The raw acoustic signal (e.g., the presented sentence) was passed through 24 bandpass filters with an equivalent rectangular bandwidth of 1 and center frequencies of 100 Hz to 4 kHz, representing the filter bank. Each filtered output was full-wave rectified and power-law compressed (i.e., the absolute value was raised to a power of .6 based on the approach by (Biesmans et al., 2017; Oderbolz et al., 2024; Schmitt et al., 2022). The power-law compression mimics the compression response of the inner ear, enabling the ear to perceive a large dynamic range of auditory input. A study by Fuglsang et al. (2020) argued that hearing impairment might change inner ear compression and therefore conducted several stimulus-response analyses across varying compression factors (from .1 to 1 in tenth increments), concluding equivalent results across the varying factors. Therefore, we chose to use a factor of .6, according to other studies conducted in our research group. The generated sub-band envelopes were then combined into an average envelope and finally resampled at 128 Hz, band-pass filtered between 2 and 8 Hz (optimal range for temporal modulations in the auditory system according to Poeppel & Assaneo, 2020) and cut to 3–5 s, matching the EEG signal.

### Neural Speech Tracking

The synchronization between auditory cortex activity and speech envelope was quantified by cross-correlation. The cross-correlation function expresses the similarity between two signals concerning a time lag, with values towards ±1 indicating perfect positive or negative correlation and values towards 0 indicating no correlation. Cross-correlation coefficients were calculated for each trial of all three modalities (AB, AVB and VO) across each channel and the corresponding speech envelopes. We decided on three topographical clusters containing a right- (1-A26, 1-A27, 1-A28, 1-A29, 1-B6, 1-B7, 1-B8, 1-B9, 1-B10, 1-B11, 1-B12) and a left-hemispheric temporo-parieto-occipital (1-A9, 1-A10, 1-A11, 1-A12, 1-A13, 1-A14, 1-A15, 1-A16, 1-D30, 1-D31, 1-D32) as well as a fronto-central (1-B31, 1-B32, 1-C1, 1-C2, 1-C3, 1-C4, 1-C11, 1-C12, 1-C13, 1-C20, 1-C21, 1-C22, 1-C23, 1-C24, 1-C25, 1-C26, 1-C27, 1-C32, 1-D1, 1-D2, 1-D3, 1-D4, 1-D12, 1-D13, 1-D18, 1-D19) cluster. The chosen electrode clusters are visualized in Figure 4C and marked with dark circles. The clusters were defined by the topography and time course of the grand average cross-correlation signal of the two auditory modalities and adapted from the approach of Schmitt et al. (2022) (Figure 4B). For a control condition, randomly selected speech envelopes were correlated with the EEG signal and compared with the experimental modalities. The comparison between both auditory experimental modalities and the control condition was done for time lags ranging from 0 to 300 ms since this time window contains two noticeable positive peaks in the cross-correlation function (Braiman et al., 2018; Horton et al., 2013; Zoefel & VanRullen, 2016). To identify significant time lags, we ran paired-sample *t*-tests between the grand average cross-correlation function of each of the auditory modalities and the control condition (Bonferroni corrected alpha level of 0.0002, 246 tests in total: for each modality and each cluster). The cross-correlation coefficients across the significant time lags and within the cluster were averaged, resulting in the average cross-correlation coefficient for each trial, in each cluster, across each modality for each participant. We additionally included a visual-only modality, which served as a control condition to test if the potentially observed visually induced increase in neural speech tracking was reflecting multisensory integration rather than the mere addition of both sensory streams. Therefore, identical electrode-cluster and time-windows were chosen to extract trial-based cross-correlation coefficients for the VO modality to then use for the statistical analysis. The average cross-correlation function of each modality and cluster can be found in Figure 4A with significant time lags highlighted and reported in the results section. These time windows provide the base for all further analyses related to neural speech tracking. Statistical tests were performed in R, version 4.1.2 (R C Team, 2020).

**Figure 3.**
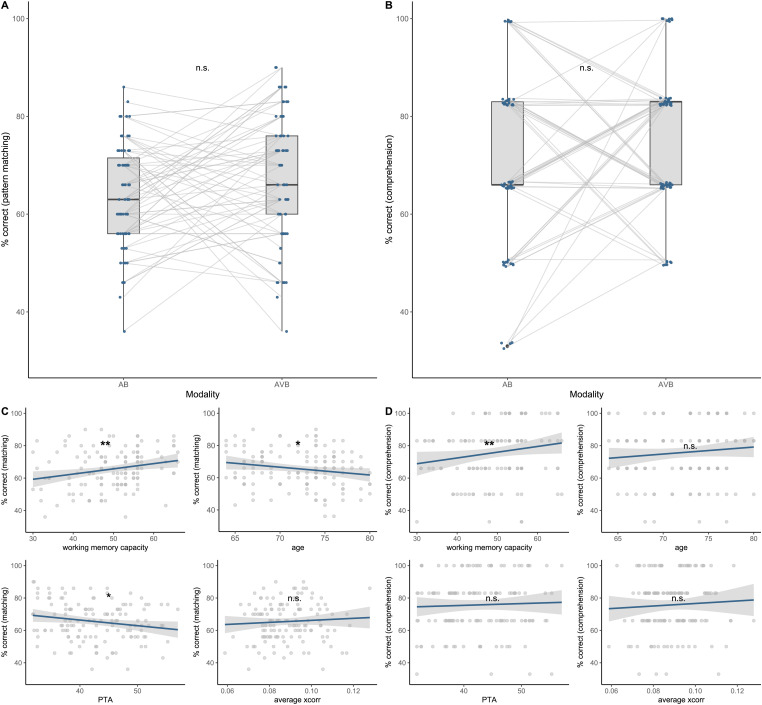
Speech in noise performance estimated by speech presentation modality, working memory capacity, age, speech tracking and hearing loss. A: No significant increase in pattern matching across the two speech presentation modalities was revealed by the model. B: No significant increase in comprehension performance across modalities was observed. Compared to pattern matching, for the comprehension task only after every 5^th^ trial, a comprehension question was asked, resulting in a six-level classification of performance (30 trials within each modality were presented in total). C: Working memory capacity was significantly positively associated with pattern matching, while age and hearing loss revealed the opposite relationship. Neural speech tracking was not significantly associated. D: Working memory capacity was significantly associated with comprehension performance, while neither age, neural speech tracking nor hearing loss explained variance in the comprehension performance. n.s. = not significant, *p < .05, **p < .01, ***p < .001.

**Figure 4.**
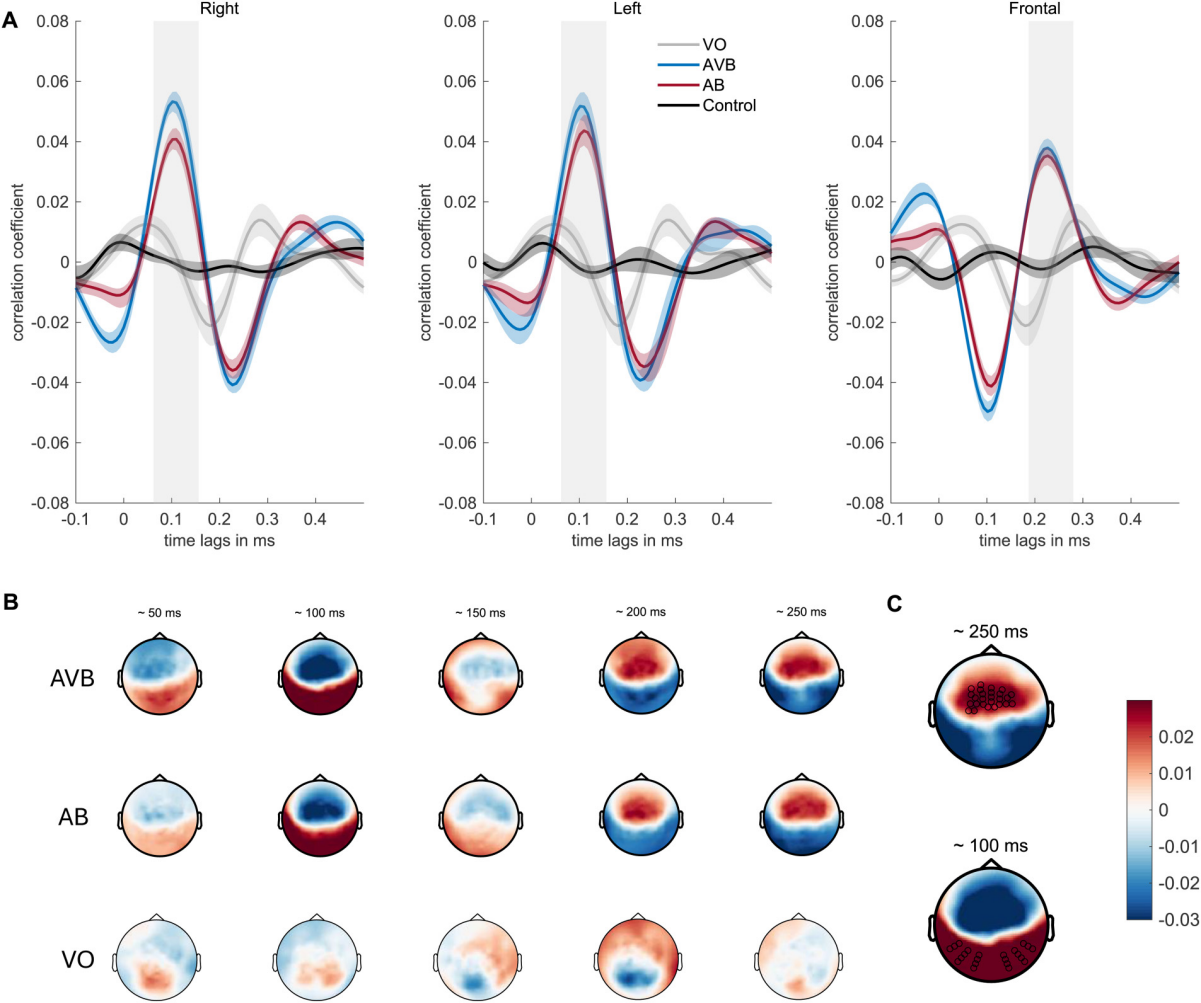
**Topographic distribution and time course of neural speech tracking**. A: Grand average cross-correlation functions of the right, the left and the frontal cluster. Significant time windows are marked as bars over the function. B: Topographic distribution and time course of the grand average cross-correlation in all three listening modalities from approximately 50 to 250 ms. C: Topographical distribution of the grand-average cross-correlation at the peaks at approximately 100 and 250 ms. Selected electrode clusters are marked with “•”. Warm colors denote positive- and cool colors negative correlations.

### Statistical Analysis: Speech in Noise Performance

To explain variance in speech in noise performance, two generalized mixed models (GLMMs) were fitted (for pattern matching and comprehension, further referred to one model, since the specifications were identical). The behavioral performance was specified as a binomial outcome (1 = correct answer, 0 = incorrect answer). The GLMM was estimated with a logistic link function from the *lme4* package (Bates et al., 2014). The model contained fixed effects of modality (categorical variable with two levels: audio-babble (AB), audio-visual babble (AVB)), as well as working memory capacity, age, and PTA (averaged across frequencies) as continuous predictors (all z-standardized), and we included a two-way interaction between modality and working memory capacity. Additionally, the model contained by-subject and by-item random intercepts and random slopes (representing the full random effect structure). A model with a maximum random effect structure was estimated (Barr et al., 2013), while iteratively adjusting the structure to avoid overparameterization, non-convergence and a singular fit. This process includes estimating a model with a random effect structure and reducing this structure (by first omitting interactions across random effects and subsequently omitting random slopes with the lowest explanation of variance) until a non-singular fit is reached. The final models are described in the results section, accompanied by detailed estimates for the individual terms. The model with the maximum random effect structure had the following specifications (using the formula notation in R):

Response∼modality*working memory capacity + *age* + *PTA* + *(modality* | *subject)* + (modality*working memory capacity|*item)*

Since interactions were included in the model, orthogonal sum-to-zero coding was used. The main effects were accordingly estimated at the grand-mean level and interpreted as such. Statistical inference was determined using likelihood ratio tests, comparing an encompassing model with a reduced model. The final model identified by this step-down process was fitted using restricted maximum likelihood. Degrees of freedom for *t*-tests and associated *p*-values were estimated using Satterthwaite's method for approximation.

### Statistical Analyses: Neural Speech Tracking

To estimate how speech presentation modality may affect neural speech tracking and whether this relationship differs across clusters, a linear mixed model (LMM) with cross-correlation as a continuous outcome variable was fitted. The model contained fixed effects of modality (categorical variable with two levels: audio-babble (AB), audio-visual babble (AVB)), fixed effects of cluster (categorical variable with three levels: fronto-central (fronto), right temporo-parieto-occipital (right) and left temporo-parieto-occipital (left) cluster), the interaction between modality and cluster, as well as working memory capacity, age, and PTA (all z-standardized) as continuous predictors. Additionally, the model contained by-subject and by-item random intercepts and random slopes (representing the full random effect structure). The model was again fitted with a maximum random effect structure and iteratively adjusted to avoid overparameterization, non-convergence and a singular fit (same procedure as for the above-described GLMM). The final model is described in the results section, accompanied by detailed estimates for the individual terms. The maximum model had the following specifications (using the R formula notation):

*Cross-correlation*∼modality*cluster + *working memory* + *age* + *PTA* + (modality*cluster | *subject)* + (modality*cluster|*item)*

Since interactions were included in the model, orthogonal sum-to-zero coding was again used (Singmann & Kellen, 2019). Statistical interference was determined using likelihood ratio tests, comparing an encompassing model with a reduced model. The final model identified by this step-down process was fitted using restricted maximum likelihood. Degrees of freedom for *t*-tests and associated *p*-values were estimated using Satterthwaite's method for approximation.

### Statistical Analyses: Multisensory Integration

To investigate if a potential increase in speech envelope tracking in AVB reflected bimodal integration rather than unimodal addition of responses, a linear mixed model was computed to estimate if cross-correlation coefficients from AVB differed significantly from the summed coefficients of AB and VO. As a first step, the algebraic sum of AB and VO ([A + V]) had to be calculated. Due to the trial-based inspection of the EEG data and the correspondingly varying rejection of trials, cross-correlation coefficients were averaged across trials, resulting in an average coefficient for each participant, in each modality and across each cluster. The across-trial averaged coefficients of AB and VO were summed for each participant in each cluster, resulting in an additional (artificial) modality A + V. The LMM contained the averaged cross-correlation coefficients as a continuous outcome variable, fixed effects of modality (categorical variable with three levels: AVB, AB, A + V) and cluster (categorical variable with three levels: fronto, left and right), as well as by-subject random intercepts. An identical model fitting procedure as described above was used. The maximum model had the following specifications:

*Average cross-correlation∼modality* + *cluster* + *(modality* | *subject)*

For consistency, orthogonal sum-to-zero coding was again used, despite the absence of interaction terms. Statistical interference was determined using likelihood ratio tests, comparing an encompassing model with a reduced model. The final model identified by this step-down process was fitted using restricted maximum likelihood. Degrees of freedom for *t*-tests and associated *p*-values were estimated using Satterthwaite's method for approximation.

## Results

### Speech in Noise Pattern Matching and Comprehension

Participants performed a pattern matching and a comprehension task, which was used to quantify speech in noise performance. To ensure that the performance was above chance we conducted one-sample *t*-tests against a 50% chance level in both speech presentation modalities. Pattern matching scores were significantly above chance in both modalities (AB: *t*(66) = 10.48, *p *< .001; AVB: *t*(66) = 11.01, *p *< .001) with a mean pattern matching performance of 63.58% (±1.29% *SEM*) in AB and 67.38% (±1.58% *SEM*) in AVB. The same procedure was applied to the comprehension task, showing performance to be significantly above chance (AB: *t*(66) = 8.98 *p *< .001; AVB: *t*(66) = 14.75, *p *< .001) with a mean comprehension performance of 72.31% (±2.48% *SEM*) in AB and 78.28% (±1.91% *SEM*) in AVB. The distribution of the speech in noise performance within the pattern matching- and comprehension task as well as individual trajectories across speech presentation modality are visualized in Figure 3A and 3B.

To estimate the contribution of speech presentation modality, working memory capacity, age, and hearing loss towards speech in noise performance, two generalized mixed models with binomial outcome measures (1 = correct, 0 = incorrect) were calculated. The models included by-subject and by-item random effects (for a detailed description of model specification go to *statistical analysis*). Due to possible higher-order effects (e.g., interactions), the predictors were estimated using an orthogonal sum-to-zero coding scheme (as opposed to a contrast coding scheme such as treatment coding). Accordingly, the main effects were estimated and interpreted at the grand mean level. Furthermore, the GLMMs were used to investigate if speech in noise performance was associated with neural speech tracking. Therefore, the models included averaged cross-correlation coefficients as a predictor. We used averaged data instead of trial-based data, as this would have biased the behavioral outcomes. While we do have complete data for the pattern matching and comprehension task (each participant answered each question), we do not have a cross-correlation coefficient for each trial, due to some trial rejection during the pre-processing of the EEG data. Therefore, cross-correlation coefficients were averaged within each cluster, within each condition, for each participant across each trial. This resulted in a total of six average cross-correlation coefficients per participant, reflecting the average tracking response across AB and AVB within both lateral-, and the frontal-cluster.

First, the pattern-matching performance was estimated by speech presentation modality, average cross-correlation coefficients, working memory capacity, age, and hearing loss. None of the interactions between the fixed effects contributed to the explained variance in speech in noise pattern matching (*p *> .05) and were thereby excluded. Likelihood ratio tests revealed a significant main effect of working memory capacity (Δχ^2^(1) = 10.391, *p *= .001), while modality was not significantly associated with pattern matching (Δ*χ*^2^(1) = 1.928, *p *= .165). A one-unit change in WMC (i.e., +1 standard deviation) is associated with 16% increase in the odds of correctly completing the pattern matching (indicated in the odds ratio, see Table 2). Furthermore, the model revealed a significant main effect of age (Δ*χ*^2^(1) = 4.699, *p *= .032), indicating a decrease in performance with enhancing age and a significant effect of PTA (Δ*χ*^2^(1) = 3.904, *p *= .045), indicating decreased performance with increasing hearing loss (see Figure 3C). Average cross-correlation coefficients were not significantly associated with the pattern-matching performance (Table 3).

**Table 2.** GLMM Estimates for Pattern Matching as Outcome variable. Final Model Configuration: Pattern Matching Response∼Modality + Xcorr.avg + WMC + PTA + age + (Modality|Subject) + (Modality + PTA|Trial).

Predictors	Estimate	*SE*	Odds Ratio	*p*
Intercept	0.73	0.07	2.01	<.001
Modality (AB)	−0.10	0.07	0.90	.162
Xcorr.avg	−0.02	0.03	0.98	.607
WMC	0.15	0.05	1.16	.001
PTA	−0.09	0.05	0.91	.045
Age	−0.10	0.05	0.81	.027

**Table 3.** GLMM Estimates for Comprehension as Outcome variable. Final Model Configuration: Comprehension Response∼Modality + Xcorr.avg + WMC + PTA + age + (Modality|Subject) + (1|Trial).

Predictors	Estimate	*SE*	Odds ratio	*p*
Intercept	1.18	0.19	6.22	<.001
Modality (AB)	−0.19	0.16	0.82	.226
Xcorr.avg	0.01	0.03	1.01	.977
WMC	0.43	0.16	1.53	.009
PTA	0.09	0.16	1.09	.587
Age	0.15	0.17	1.16	.362

Second, comprehension performance was estimated by speech presentation modality, averaged cross-correlation coefficients, working memory capacity, age, and hearing loss. Again, none of the interactions between the fixed effects contributed to the explained variance in speech in noise comprehension performance (*p *> .05) and were thereby excluded. The likelihood ratio test revealed a significant main effect of working memory (Δ*χ*^2^(1) = 6.542, *p *= .011), indicating a positive association between working memory capacity and comprehension performance. A one-unit change in WMC (i.e., +1 standard deviation) is associated with 53% increase in the odds of answering correctly in a comprehension task (see Figure 3D). It should be mentioned here that comprehension was quantified over 6 items (after every fifth pattern-matching task, another comprehension question was asked), resulting in a six-level classification of performance (see Figure 3B). Accordingly, the degree of increase in the odds of answering correctly appears much greater compared to the model for pattern matching (17% increase in the odds with one unit change in WMC). As for speech modality, the model revealed no significant main effect (Δ*χ*^2^(1) = 1.399, *p *= .237), indicating that speech in noise comprehension performance does not significantly improve with congruent visual speech cues. Furthermore, no significant association between comprehension performance and age, average cross-correlation coefficients or hearing loss (*p *> .362) was revealed.

### Neural Speech Tracking: Significant Time-Windows

The cortical representation of speech was quantified by the cross-correlation between the EEG time series and the amplitude envelope of the speech stimuli. In line with previous work (Horton et al., 2013; Zoefel & VanRullen, 2016), in our grand average cross-correlation functions for both speech presentation modalities a prominent positive peak at ∼100 ms and a later one at around ∼250 ms with an inversed polarity across the scalp was observed (Figure 4). To identify time lags with significant deviations of the cross-correlation coefficients from zero, the cross-correlation function across the two auditory modalities (AVB and AB) were compared to a control condition. A pairwise comparison revealed several significant time lags (Bonferroni corrected with an alpha level of .0002; Figure 4A). To define the significant time windows only positive deflections in the cross-correlation function and the corresponding significant time lags were considered (Abrams et al., 2008; Braiman et al., 2018), since values converging towards 1 reflect a positive linear relationship between the two-time series. We also extracted the cross-correlation coefficients corresponding to the negative peaks to conduct an additional analysis investigating if negative linear relationships were observable across the negative cross-correlation coefficients. The additional analysis revealed no significant difference in neural speech tracking across modalities when represented by negative correlation coefficients. In the left temporo-parieto-occipital electrode cluster significant lags were found from 62 to 164 ms for AB and from 55 to 150 ms for AVB. In the right temporo-parieto-occipital cluster, lags from 55 to 164 ms appeared to be significant for AB, whereas lags from 54 to 156 ms were significant for AVB. In the fronto-central cluster, time lags from 180 to 296 ms were significant for AB whereas lags ranging from 180 to 288 ms were significant for AVB. To compare the neural speech tracking response in AVB with the cumulated response of AB and VO, the same clusters and significant time lags as for AVB were used.

### Neural Speech Tracking: Multisensory Integration

A hierarchical approach was chosen to investigate if potential differences in speech envelope tracking across modalities reflected bimodal integration rather than unimodal addition of tracking responses. To this end, a mixed model was computed to estimate if cross-correlation coefficients from AVB differed significantly from the summed coefficients of AB and VO. Due to the trial-based inspection of the EEG data and the correspondingly varying rejection of trials, cross-correlation coefficients were averaged across trials, resulting in an average coefficient for each participant, in each modality and across each cluster. These average coefficients were used to calculate the algebraic sum of AB and VO ([A + V]), which served as an additional level of the factor modality. Likelihood ratio tests indicate a significant main effect of modality (Δ*χ*^2^(2) = 13.367, *p* = .001), indicating a significant difference in averaged cross-correlation coefficients across modalities. Post-hoc pairwise comparisons revealed a significant increase in neural speech tracking in AVB compared to AB (AB – AVB: Δ*β* = −0.005, SE = 0.001, *t* = −3.58, *p* = .001) as well as significantly increased neural speech tracking in AVB compared to the sum of AB and VO ([A + V] − AVB: Δ*β* = −0.004, SE = 0.001, *t* = −2.57, *p* = .028). This result suggests that the observed increase in cross-correlation coefficients under audio-visual speech presentation reflects multisensory integration rather than the sum of the two independent streams of auditory and visual information. Based on this result, we further investigated the relationship between speech presentation modality and neural speech tracking.

### Neural Speech Tracking: Changes Across Modalities

**Figure 5.**
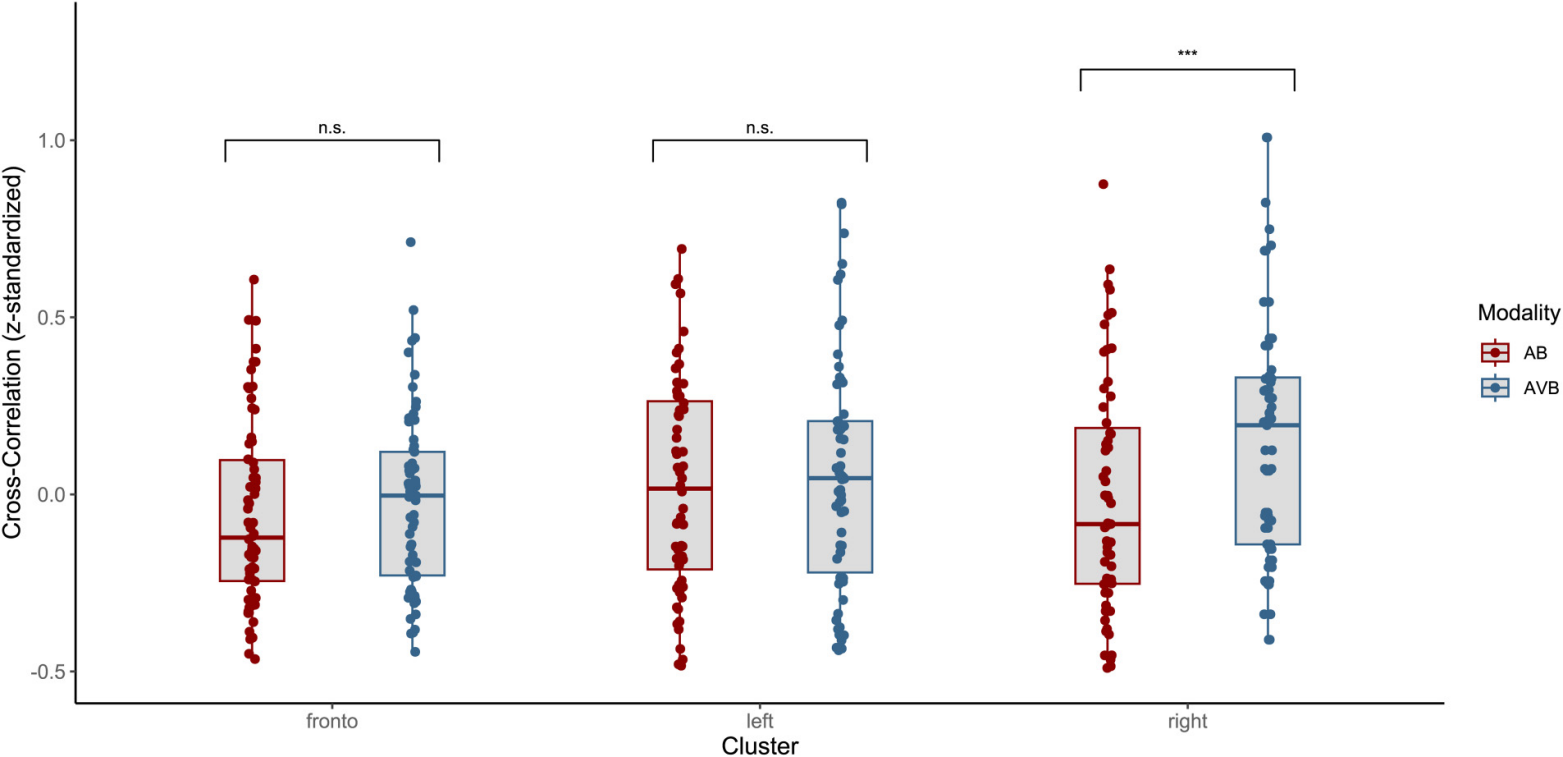
**Neural speech tracking across modalities and cluster**. Neural speech tracking significantly differed across modalities. In the presence of congruent visual speech cues, cross-correlation coefficients increased, particularly in the right auditory-related cluster. n.s. = not significant, *p < .05, **p < .01, ***p < .001.

The above-mentioned significant time windows (Figure 4A) served as a base to extract trial-level data by averaging cross-correlation coefficients for each window, within each modality and cluster. To estimate the effect of modality on neural speech tracking, an LMM was fitted whereby cross-correlation coefficients were chosen as a continuous outcome variable. While we were not particularly interested in comparing cross-correlation coefficients between the clusters directly (since they correspond to different time-windows), we were interested in investigating if potential changes in the tracking response due to the modality differed across the three clusters. Therefore, we added the factor cluster into the model, to include it in an interaction term with modality. Additionally, we also included working memory capacity, PTA, and age as fixed effects, as they might explain some variance in the cross-correlation coefficients. Furthermore, the sum-to-zero coding scheme was maintained and accordingly, the main effects were again estimated and interpreted at the grand mean level. Regarding the fixed effects, likelihood ratio tests revealed a significant main effect for modality (Δ*χ*^2^(1) = 11.151, *p *< .001), indicating significantly lower cross-correlation coefficients when the speech in noise was presented as merely auditory compared to the grand mean. Post-hoc pairwise comparison was computed, revealing a significant increase in the neural speech tracking response when presented with congruent visual speech cues (AB – AVB: Δ*β* = −0.005, *SE *= 0.001, *t *= −3.48, *p *= .001). Furthermore, a significant main effect for the cluster was revealed (Δ*χ*^2^(2) = 12.001, *p *= .002), indicating a significant difference in the neural tracking response across the clusters. Since the clusters containing cross-correlation coefficients reflecting different temporal windows, we were not interested in comparing the tracking response between those clusters directly, but rather the change in neural speech tracking across modalities within those clusters. Therefore, we included a two-way interaction between cluster and modality, to evaluate potential modality-induced differences in tracking across the cluster. The model revealed a significant contribution to the explained variance by the interaction between modality and cluster (Δ*χ*^2^(2) = 21.547, *p *< .001), indicating significant differences in the tracking response across modalities and clusters. Post-hoc pairwise comparison revealed no statistically significant difference in the tracking response within the left cluster across modalities (left_AB_ – left_AVB_: Δ*β* = −0.003, *SE *= 0.002, *t *= −1.48, *p *= .676), nor within the frontal cluster across modalities (fronto_AB_ – fronto_AVB_: Δ*β* = −0.001, *SE *= 0.002, *t *= −0.62, *p *= .991). Regarding the right cluster, the comparison revealed a significant increase in the tracking response under audio-visual speech presentation compared to a mere auditory presentation (right_AB_ – right_AVB_: Δ*β* = −0.01, *SE *= 0.002, *t *= −5.626, *p *< .001), indicating that the observed visual enhancement of neural speech tracking might rely on a specific visual enhancement within the right temporo-parieto-occipital cluster (see Figure 5). Finally, the model revealed no significant association between the individual variables working memory capacity, hearing loss nor age regarding the neural speech tracking response. The detailed model estimates are summarized in Table 4. Furthermore, we conducted the same model but with cross-correlation coefficients reflecting the negative peaks (see Figure 4), while using the opposite time-windows (the early time-window to extract coefficients from the frontal cluster, and the later time-window to extract coefficients from the lateral clusters). We used these clusters within a model with the exact same specification but were not able to establish any significant variation in neural speech tracking across modalities.

**Table 4.** Detailed Estimates From the Neural Speech Tracking Model. Neural Speech Tracking was Estimated by Speech Presentation Modality, Cluster, WMC, PTA, and Age. Final Model Configuration: Cross-Correlation Coefficient∼Modality * Cluster + WMC + PTA + age + (Modality|Subject) + (1|Trial).

Predictors	Estimate	*SE*	*df*	*t*	*p*
Intercept	0.0929	0.0018	71.19	49.83	<.001
Modality (AB)	−0.0028	0.0008	66.51	−3.47	<.001
Cluster (fronto)	−0.0022	0.0007	8377.34	−3.01	.002
Cluster (left)	−0.0001	0.0007	8378.65	−0.12	.903
WMC	0.0010	0.0013	66.85	0.79	.433
PTA	0.0002	0.0012	65.86	0.15	.879
Age	−0.0001	0.0013	66.47	−0.04	.965
Modality (AB):Cluster (fronto)	0.0021	0.0007	8377.48	2.96	.003
Modality (AB):Cluster (left)	0.0018	0.0007	8376.23	1.62	.105

## Discussion

In the present study, we investigated the impact of visual speech cues on speech perception in babble noise (quantified by a pattern matching and comprehension task) as well as neural processing of speech (quantified by speech envelope tracking) in a sample of older adults with hearing loss. Furthermore, we investigated whether the neural change in response to visual speech cues was associated with the variance in audio-visual speech perception. Our sample (*N* = 67) was instructed to decide whether sound snippets were part of the presented sentence or not (i.e., pattern-matching task), and a content-related question was asked after every fifth sentence (i.e., comprehension task). The presented speech material consisted of complex, continuous sentences, which were presented with multi-talker babble noise (SNR = 0), whereby half of the speech material was accompanied by congruent visual lip movements (audio-visual speech presentation). In parallel to the presented speech material, cortical activity was recorded using scalp EEG, from which we derived neuronal speech envelope tracking. The neuronal response was calculated using cross-correlation between the EEG signal and the speech envelope and analyzed for the models as a continuous variable.

### Understanding Continuous Speech in Babble Noise Depends on Individual Working Memory

We hypothesized increased speech in noise performance under audio-visual (AV) speech presentation and a positive relationship between working memory capacity and SiN performance. Our results partially confirm this hypothesis, as we can report a positive association between working memory capacity and performance, but no effect of presentation modality. The absence of a performance increase under AV was surprising, considering the well-established AV benefit of intelligibility under adverse listening situations for normal hearing younger- (Bernstein et al., 2004; Grant & Seitz, 2000; Ross et al., 2007; Yi et al., 2021), and older adults (Tye-Murray et al., 2016; Winneke & Phillips, 2011) as well as hearing impaired individuals (Puschmann et al., 2019; Tye-Murray et al., 2007). However, this effect is strongly based on target detection tasks, which are highly suitable to measure intelligibility. When speech is accompanied by congruent visual information, articulatory movement is provided, conveying complementary information about degraded auditory input, resulting in facilitated detection. This audio-visual benefit is often quantified through the increased signal-to-noise ratio (Bernstein et al., 2004; Grant & Seitz, 2000; Sumby & Pollack, 2005), suggesting increased intelligibility even in more adverse listening situations. We argue that the observed deviation of our results compared to the existing literature is partially based on task differences. In the current study, participants had to listen, store, and match complex continuous speech with a target stimulus. Successful completion of this task required that the auditory input was intelligible, for both pattern matching and comprehension. This pattern-matching task might not reflect a traditional measure of intelligibility and therefore does not allow to quantify if audio-visual speech cues increase perception thresholds in noise, however, this was not the intention. The aim of the study was to investigate if audio-visual speech cues facilitate comprehension of natural continuous speech, which goes beyond mere target detection and is reflected in the applied task. As such, we agree that visual speech cues facilitate target identification and therefore intelligibility in noise but argue that intelligibility does not fully reflect the demand of speech comprehension.

Understanding audio-visual speech requires sensory processing (e.g., acoustic information in speech and visual speech information in the face) and cognitive processing of the heard information, such as storing and updating within the working memory system (e.g., Akeroyd, 2008; Heald & Nusbaum, 2014; Wingfield et al., 2015). Schneider and Pichora-Fuller (2000) proposed an integrated perceptual-cognitive system, assuming a direct link between sensory processing and higher-order cognition, whereby efficient resource allocation is crucial for sufficient processing. Accordingly, if too many resources are utilized for sensory processing, higher-order processing, such as encoding the heard information, will be limited. Considering such theoretical assumptions, additional sensory information such as visual speech cues might increase the processing load further. A study by Brault et al. (2010) demonstrated improved speech intelligibility under bimodal presentation, but no improvement for word recall, suggesting no audio-visual benefit for speech comprehension, while other studies assume increased processing effort through audio-visual speech presentation (e.g., Brown & Strand, 2019; Fraser et al., 2010; Miller et al., 2017; Mishra et al., 2013; Picou et al., 2011). In the here reported results, we were not able to establish an association between visual speech presentation modality and the performance within the used speech task. However, looking at the individual trajectories, some participants show a visually induced performance increase, while others show a substantial decrease. This suggests that while some listeners benefit from additional sensory information, others seem to be challenged by it. Considering a theoretical allocation of processing resources, we argue that additional sensory information (such as visual speech cues) can increase perceptual demand, while working memory capacity might provide support for some individuals to meet this challenge. The substantial role of working memory in the context of speech perception has been demonstrated extensively (e.g., Anderson et al., 2013; Lunner, 2003; Peelle, 2018; Rabbitt, 1968; Wingfield et al., 2015; Zekveld et al., 2011). Furthermore, working memory capacity seems to partially explain individual differences in speech perception in hearing-impaired individuals (Humes et al., 2013; Rönnberg et al., 2016; Rudner et al., 2011, 2012). We argue that audio-visual benefit for continuous speech becomes more complex considering the interaction of individual sensory- and cognitive capacity. As such, working memory capacity plays a considerable role in the context of adverse listening situations and speech comprehension, while additional sensory information can provide support, but also reflect further processing challenges. However, while the used tasks in this study might reflect a more realistic demand of real-world listening, they also target working memory-related aspects, therefore partially explaining the strong association between individual working memory capacity and task performance.

### Bimodal Speech Presentation Increases Neural Envelope Tracking

We further hypothesized increased neural speech tracking under bimodal-compared to mere auditory speech presentation, which was confirmed by significantly increased cross-correlation when congruent visual speech cues were present, corroborating several previous studies for normal hearing (Aller et al., 2022; Luo et al., 2010; Mégevand et al., 2020; Micheli et al., 2018; Park et al., 2016) as well as older hearing impaired individuals (Haider et al., 2024; Puschmann et al., 2019; Wang et al., 2023). Visual speech cues appear to enhance cortical tracking of the speech envelope, particularly in noise (Crosse et al., 2015; Zion Golumbic et al., 2013), in younger and older individuals (Puschmann et al., 2019). This effect has been associated with the correlation between the articulatory movement of the lip- and jaw area with the temporal regularities of the speech envelope (Chandrasekaran et al., 2009; Hauswald et al., 2018; Lalonde & Werner, 2019; Park et al., 2016; Suess et al., 2022). Schroeder et al. (2008) proposed that articulatory movement enhances the phase-alignment of auditory cortical oscillations to the speech signal, suggesting a modulation of neural activity rather than a mere addition of response (Besle et al., 2008; Micheli et al., 2018; Park et al., 2016). The here observed AV increase in neural speech tracking was significantly higher than the combined tracking response from the visual- and the auditory-only speech presentation modality. As such, we argue that increased neural speech tracking under audio-visual speech presentation reflects a visual modulation of speech envelope tracking in noise, suggesting a multisensory integration.

Furthermore, our results revealed that a visually induced increase in neural speech tracking was particularly strong in a right hemispheric temporo-parieto-occipital cluster, representing earlier (approx. 50 to 150 ms) processing from auditory-related areas. Several studies argue that visually induced modulation of auditory speech processing occurs across multiple stages (Baart et al., 2014; Eskelund et al., 2011; Peelle & Sommers, 2015; Schwartz et al., 2004; van Wassenhove et al., 2005), suggesting an early stage, whereby visual information contributes temporal cues, and a later stage where visual cues provide information for lexical selection. The early integration of information is argued to enhance the sensitivity of the auditory cortex, which has been supported by several studies (Calvert et al., 1997; Grant & Seitz, 2000; Yamashita et al., 2013). Considering this line of evidence in the context of the here reported results, we argue that visual speech cues during natural continuous speech in noise might increase the sensitivity of auditory cortical processing for sensory information. It is important to note that, visually induced tracking increase was particularly strong in the right auditory-related cluster. Lateralisation of speech processing has frequently been investigated within the “asymmetric sampling in time” AST hypotheses (Ben Shalom & Poeppel, 2008; Poeppel, 2003), which states that the initial representation of speech is bilateral but becomes asymmetric in later processing stages (e.g., Doelling et al., 2014; Giraud & Poeppel, 2012; Liem et al., 2014; Meyer, 2008; Rufener et al., 2016). While the hypotheses consider age-typical auditory performance, Giroud et al. (2019) extended this through the perspective of aging, arguing that age-related structural degradation is counteracted by increased bilateral processing of speech, particularly through stronger involvement of right auditory-related areas. Considering our sample, we extend this argument through hearing loss. We speculate that some participants are not sufficiently recruiting both auditory areas (left as well as right) to overcome partly age- and partly sensory-related decline in auditory networks, resulting in the observed increase of envelope tracking in the right auditory cluster. The presence of congruent visual speech cues might provide an additional source of information that promotes bilateral recruitment for neural speech processing.

### Neural Envelope Tracking is not Associated With Speech in Noise Comprehension

Lastly, we hypothesized a positive relationship between neural speech tracking and speech in noise performance, which has not been confirmed by our results. Several empirical evidence reports a positive relationship between speech tracking and speech comprehension and interprets this as functional (Gillis et al., 2021; Kurthen et al., 2021). However, other evidence interprets increased tracking in older individuals as an expression of speech comprehension difficulties. Decruy et al. (2019) investigated speech envelope tracking in speech in noise in older, middle-aged, and younger adults. While they report that at the individual level, speech tracking increases when the speech signal is better understood, they also show that at the group level, older adults show increased speech tracking despite performing at the same level as younger adults. The authors argue that increased tracking does not reflect better speech understanding but rather the difficulties experienced with speech in noise perception. Furthermore, increased neural speech tracking could also represent the inefficient use of cognitive resources (Presacco et al., 2016). The authors argue that neural speech tracking represents an overrepresentation of the speech signal, which is particularly evident in older age and hearing loss (e.g., Goossens et al., 2018). This overrepresentation expresses an imbalance between excitatory and inhibitory mechanisms, which leads to inefficient the utilization of neuronal resources. This argument is supported by the fact that with increasing age, a reduction in cortical network connectivity can be observed, which leads to neighboring cortical regions processing the same stimulus independently (i.e., inefficiently, redundantly) instead of collaboratively (Peelle & Sommers, 2015). Following this line of reasoning, we argue that the increase in neural speech tracking may reflect over-representative sensory processing and might not be functionally related to speech comprehension.

## Limitations

The aim of this study was to investigate if congruent visual speech cues facilitate speech in noise comprehension in older hearing-impaired individuals. As such, we presented naturalistic continuous sentences in babble noise and assessed speech comprehension through content-related questions and a pattern matching task. We were not able to demonstrate visually induced improvement in speech comprehension, which was surprising given the existing literature. While we argued that this effect is strongly based on target stimuli detection, we have to acknowledge that our task might not have been ideal to assess speech comprehension sufficiently. The strong association with working memory capacity is not surprising, given the task nature and the speech material complexity. Nevertheless, we argue that the investigation of audio-visual benefits regarding the comprehension of spoken communication requires the consideration of sensory as well as cognitive contributions, to fully understand how visual speech cues facilitate this process. For future research, we suggest the careful consideration of both sensory- and cognitive-processing demands of speech. The development of naturalistic tasks to assess speech perception becomes highly important in order to investigate if audio-visual benefit goes beyond speech recognition.

## Conclusion

This study investigated behavioral and neural processing of natural continuous speech in babble noise as a function of varying speech presentation modalities in older hearing-impaired individuals. Our data revealed that behavioral speech perception did not differ substantially between mere auditory- and audio-visual speech presentation modalities, while the change in performance was characterized by large individual variance. Additionally, the data revealed a strong association between individual working memory capacity and task performance, suggesting a more complex interplay between sensory and cognitive processing in the context of natural continuous speech. However, the used tasks targeted specifically working memory-related abilities, therefore explaining this association partially. Nevertheless, we strongly recommend considering individual cognitive capacities when investigating speech perception. Furthermore, we report that visual speech information was accompanied by increased cortical tracking of the speech envelope, although this increase does not reflect better speech understanding. This increase was particularly found in a right-hemispheric auditory topographical cluster, suggesting that the presence of congruent visual speech cues may provide an additional source of information that elicits bilateral recruitment of neural speech processing in older individuals with hearing loss.
